# Supplementary material for: Literature Review: Evaluation of Drug Removal Techniques in Municipal and Hospital Wastewater
Source: Int J Environ Res Public Health. 2022 Oct 12;19(20):13105. doi: 10.3390/ijerph192013105 (PMC9602914; doi:10.3390/ijerph192013105)
Supplement: Supplementary file 1 [file ijerph-19-13105-s001.zip › ijerph-1892410-supplementary.pdf]

Supplemental Information

Table S1. Set of studies included in the systematic review.

Code	Database	Scientific journal	Quartile SJR	Title	Country	Ref.	DOI
M01	Scopus	Water Research	Q1	Nanofiltration as tertiary treatment method for removing trace pharmaceutically active compounds in wastewater from wastewater treatment plants	Spain	[27]	https://doi.org/10.1016/j.watres.2017.08.070
M02	Scopus	Applied Catalysis B: Environmental	Q1	Removal of antibiotics, antibiotic-resistant bacteria and their associated genes by graphene-based TiO_2 composite photocatalysts under solar radiation in urban wastewaters	Cyprus	[28]	https://doi.org/10.1016/j.apcatb.2017.11.020
M03	Scopus	Journal of Water Reuse and Desalination	Q1	Evaluating occurrence of contaminants of emerging concerns in MF/RO treatment of primary effluent for water reuse – Pilot study	USA	[29]	https://doi.org/10.2166/wrd.2019.004
M04	Scopus	International Journal of Environmental Research and Public Health	Q1	Removal of Pharmaceutical and Personal Care Products (PPCPs) from Municipal Waste Water with Integrated Membrane Systems, MBR-RO/NF	China	[30]	https://doi.org/10.3390/ijerph15020269
M05	Scopus	Water (Switzerland)	Q1	WWTP Effluent Quality Improvement for Agricultural Reuse Using an Autonomous Prototype	Spain	[31]	https://doi.org/10.3390/w12082240
M06	Proquest	Water Science and Technology	Q2	Occurrence and elimination of antibiotics in three sewage treatment plants with different treatment technologies in Urumqi and Shihezi, Xinjiang	China	[32]	https://doi.org/10.2166/wst.2017.013
M07	Proquest	Environmental Science and Pollution Research	Q1	Occurrence and behavior of pharmaceutical compounds in a Portuguese wastewater treatment plant: Removal efficiency through conventional treatment processes	Portugal	[33]	https://doi.org/10.1007/s11356-017-9012-7
M08	Proquest	Environmental Science and Pollution Research	Q1	Ozonation in advanced treatment of secondary municipal wastewater effluents for the removal of micropollutants	Turkey	[34]	https://doi.org/10.1007/s11356-020-10339-5
M09	Proquest	Water (Switzerland)	Q1	Towards the Removal of Antibiotics Detected in Wastewaters in the POCTEFA Territory: Occurrence and TiO_2 Photocatalytic Pilot-Scale Plant Performance	Spain	[35]	https://doi.org/10.3390/w12051453
M10	Proquest	Water Science and Technology	Q2	High-rate algal pond for removal of pharmaceutical compounds from urban domestic wastewater under tropical conditions. Case study: Santiago de Cali, Colombia	Colombia	[36]	https://doi.org/10.2166/wst.2020.362

M11	Proquest	Membranes	Q2	Occurrence of Antibiotics, Antibiotic Resistance Genes and Viral Genomes in Wastewater Effluents and Their Treatment by a Pilot Scale Nanofiltration Unit	Portugal	[39]	https://doi.org/10.3390/membranas11010009
M12	Ebsco host	Molecules	Q1	A Survey of the Presence of Pharmaceutical Residues in Wastewaters. Evaluation of Their Removal using Conventional and Natural Treatment Procedures	Spain	[41]	https://doi.org/10.3390/molecules25071639
M13	Sciencedirect	Journal of Hazardous Materials	Q1	Fate of priority pharmaceuticals and their main metabolites and transformation products in microalgae-based wastewater treatment systems	Spain	[45]	https://doi.org/10.1016/j.jhazmat.2019.121771
M14	Sciencedirect	Chemosphere	Q1	Fate of active pharmaceutical ingredients in a northern high-rate algal pond fed with municipal wastewater	Sweden	[46]	https://doi.org/10.1016/j.chemosphere.2021.129763
M15	Sciencedirect	Emerging Contaminants	Q1	Assessment of non-steroidal anti-inflammatory drugs from selected wastewater treatment plants in Southwestern India	India	[47]	https://doi.org/10.1016/j.emcon.2021.01.001
M16	Sciencedirect	Science of The Total Environment	Q1	Occurrence and environmental impact of pharmaceutical residues from conventional and natural wastewater treatment plants in Gran Canaria (Spain)	Spain	[48]	https://doi.org/10.1016/j.scitotenv.2017.05.058
M17	Sciencedirect	Chemosphere	Q1	Comparison of different advanced treatment processes in removing endocrine disruption effects from municipal wastewater secondary effluent	China	[49]	https://doi.org/10.1016/j.chemosphere.2016.10.031
M18	Sciencedirect	Chemical Engineering Journal	Q1	Removal of emerging contaminants from a primary effluent of municipal wastewater by means of sequential biological degradation-solar photocatalytic oxidation processes	Spain	[50]	https://doi.org/10.1016/j.cej.2016.01.022
M19	Sciencedirect	Chemical Engineering Journal	Q1	Pharmaceutical biological degradation, sorption and mass balance determination in a conventional activated-sludge wastewater treatment plant from Murcia, Spain	Spain	[51]	https://doi.org/10.1016/j.cej.2017.01.048
M20	Sciencedirect	Journal of Water Process Engineering	Q1	Fate evaluation of pharmaceuticals in solid and liquid phases at biological process of full-scale municipal wastewater treatment plants	South Korea	[52]	https://doi.org/10.1016/j.jwpe.2021.102538
M21	Sciencedirect	Science of The Total Environment	Q1	Assessing membrane fouling and the performance of pilot-scale membrane bioreactor (MBR) to treat real municipal wastewater during winter season in Nordic regions	Finland	[53]	https://doi.org/10.1016/j.scitotenv.2016.11.122
H01	Scopus	Environmental Research	Q 1	Assessment of multiclass pharmaceutical active compounds (PhACs) in hospital WWTP influent and effluent samples by UHPLC-Orbitrap	Greece	[23]	https://doi.org/10.1016/j.envres.2020.110152

				MS: Temporal variation, removals, and environmental risk assessment			
H02	Scopus	Journal of Toxicology - Hindawi	Q2	Assessment of Pharmaceuticals, Personal Care Products, and Hormones in Wastewater Treatment Plants Receiving Inflows from Health Facilities in North West Province, South Africa	South Africa	[24]	https://doi.org/10.1155/2018/3751930
H03	Scopus	Chemical Engineering Journal	Q1	Removal of pharmaceuticals, toxicity, and natural fluorescence through the ozonation of biologically-treated hospital wastewater, with further polishing via a suspended biofilm	Denmark	[25]	https://doi.org/10.1016/j.cej.2018.11.112
H04	Scopus	Environmental Science and Pollution Research	Q1	Investigating the removal of some pharmaceutical compounds in hospital wastewater treatment plants operating in Saudi Arabia	Saudi Arabia	[26]	https://doi.org/10.1007/s11356-016-6389-7
H05	Proquest	Environmental Monitoring and Assessment	Q2	Occurrence of antibiotics residues in hospital wastewater, wastewater treatment plant, and surface water in Nairobi County, Kenya	Kenya	[37]	https://doi.org/10.1007/s10661-019-7952-8
H06	Proquest	Archives of Environmental Contamination and Toxicology	Q2	QuEChERS Approach for the Analysis of Three Fluoroquinolone Antibiotics in Wastewater: Concentration Profiles and Ecological Risk in Two Nigerian Hospital Wastewater Treatment Plants	Nigeria	[38]	https://doi.org/10.1007/s00244-020-00789-w
H07	Proquest	Environmental Science and Pollution Research	Q1	Non-antimicrobial pharmaceuticals can affect the development of antibiotic resistance in hospital wastewater	Slovakia	[40]	https://doi.org/10.1007/s11356-020-07950-x
H08	Ebsco host	Archives of Environmental Contamination and Toxicology	Q2	Analysis, Occurrence, and Ecological Risk Assessment of Diclofenac and Ibuprofen Residues in Wastewater from Three Wastewater Treatment Plants in South-Western Nigeria	Nigeria	[42]	https://doi.org/10.4314/jasem.v25i3.5
H09	Ebsco host	Chemical Engineering Communications	Q2	Photocatalytic degradation of ciprofloxacin antibiotic by TiO_2 nanoparticles immobilized on a glass plate	Iran	[43]	https://doi.org/10.1080/00986445.2019.1573168
H10	Sciencedirect	Environmental Pollution	Q1	Determination of metals and pharmaceutical compounds released in hospital wastewater from Toluca, Mexico, and evaluation of their toxic impact	Mexico	[44]	https://doi.org/10.1016/j.envpol.2018.04.116
